# Supplementary material for: Iterative improvement in the automatic modular design of robot swarms
Source: PeerJ Comput Sci. 2020 Dec 7;6:e322. doi: 10.7717/peerj-cs.322 (PMC7924708; doi:10.7717/peerj-cs.322)
Supplement: Supplemental Information 3 [file peerj-cs-06-322-s003.zip › argos3/doc/api/standalone/a00491_source.html]

ARGoS: plugins/robots/generic/control\_interface/ci\_colored\_blob\_perspective\_camera\_sensor.h Source File


- Main Page
- Related Pages
- Namespaces
- Classes
- Files

- File List
- File Members

# plugins/robots/generic/control\_interface/ci\_colored\_blob\_perspective\_camera\_sensor.h

Go to the documentation of this file.

```
00001 #ifndef CI_COLORED_BLOB_PERSPECTIVE_CAMERA_SENSOR_H
00002 #define CI_COLORED_BLOB_PERSPECTIVE_CAMERA_SENSOR_H
00003 
00004 namespace argos {
00005    class CCI_ColoredBlobPerspectiveCameraSensor;
00006 }
00007 
00008 #include <argos3/core/control_interface/ci_sensor.h>
00009 #include <argos3/core/utility/math/angles.h>
00010 #include <argos3/core/utility/datatypes/color.h>
00011 #include <vector>
00012 
00013 #include <argos3/core/utility/logging/argos_log.h>
00014 
00015 namespace argos {
00016 
00026    class CCI_ColoredBlobPerspectiveCameraSensor: public CCI_Sensor {
00027 
00028    public:
00029 
00040       struct SBlob {
00041          /* Color */
00042          CColor Color;
00043          /* Blob position */
00044          SInt32 X, Y;
00048          SBlob() :
00049             Color(CColor::BLACK),
00050             X(0), Y(0) {
00051          }
00055          SBlob(const CColor& c_color,
00056                SInt32 n_x,
00057                SInt32 n_y) :
00058             Color(c_color),
00059             X(n_x),
00060             Y(n_y) {
00061          }
00062 
00063          friend std::ostream& operator<<(std::ostream& c_os, const SBlob& s_blob) {
00064             c_os << "(Color = " << s_blob.Color << "," << "X = " << s_blob.X << ",Y = " << s_blob.Y << ")";
00065             return c_os;
00066          }
00067       };
00068 
00072       typedef std::vector<SBlob*> TBlobList;
00073 
00079       struct SReadings {
00080          TBlobList BlobList;
00081          UInt64 Counter;
00082 
00083          SReadings() :
00084             Counter(0) {
00085          }
00086 
00087          friend std::ostream& operator<<(std::ostream& c_os, const SReadings& s_reading) {
00088             c_os << "Counter: " <<  s_reading.Counter << std::endl;
00089             for (size_t i = 0; i < s_reading.BlobList.size(); i++) {
00090                c_os << "Blob[" << i << "]: " << s_reading.BlobList[i] << std::endl;
00091             }
00092             return c_os;
00093          }
00094       };
00095 
00096    public:
00097 
00101       CCI_ColoredBlobPerspectiveCameraSensor() {}
00102 
00106       virtual ~CCI_ColoredBlobPerspectiveCameraSensor() {}
00107 
00112       const SReadings& GetReadings() const;
00113 
00117       virtual void Enable() = 0;
00118 
00122       virtual void Disable() = 0;
00123 
00124 #ifdef ARGOS_WITH_LUA
00125       virtual void CreateLuaState(lua_State* pt_lua_state);
00126 
00127       virtual void ReadingsToLuaState(lua_State* pt_lua_state);
00128 #endif
00129 
00130    protected:
00131 
00132       SReadings m_sReadings;
00133 
00134    };
00135 
00136 }
00137 
00138 #endif
```

---

Generated on 10 Jul 2018 for ARGoS by 
 1.6.1
